# Association between Dysphagia and Frailty in Older Adults: A Systematic Review and Meta-Analysis

**DOI:** 10.3390/nu14091812

**Published:** 2022-04-27

**Authors:** Ru-Yung Yang, An-Yun Yang, Yong-Chen Chen, Shyh-Dye Lee, Shao-Huai Lee, Jeng-Wen Chen

**Affiliations:** 1 Graduate Institute of Business Administration, Fu Jen Catholic University, New Taipei City 242062, Taiwan; 109087@gapps.nou.edu.tw; 2 Department of Otolaryngology-Head and Neck Surgery, Cardinal Tien Hospital and School of Medicine, Fu Jen Catholic University, New Taipei City 23148, Taiwan; a75886@gmail.com; 3 Master Program of Big Data in Biomedicine, College of Medicine, Fu Jen Catholic University, New Taipei City 242062, Taiwan; yongchenchen0824@gmail.com; 4 School of Medicine, College of Medicine, Fu Jen Catholic University, New Taipei City 242062, Taiwan; 5 Fu Jen Affiliated Clinics, Fu Jen Catholic University Hospital, New Taipei City 242062, Taiwan; shyhdye@ntunhs.edu.tw; 6 Graduate Program of Long-Term (Custodial) Care, College of Medicine, Fu Jen Catholic University, New Taipei City 242062, Taiwan; 7 Department of Oral Hygiene and Healthcare, Cardinal Tien Junior College of Healthcare and Management, New Taipei City 23143, Taiwan; sango@ctcn.edu.tw; 8 Department of Medical Education and Research, Cardinal Tien Hospital, New Taipei City 23148, Taiwan; 9 Department of Otolaryngology-Head and Neck Surgery, National Taiwan University Hospital, Taipei 100225, Taiwan

**Keywords:** dysphagia, deglutition, frailty, prefrailty, aging

## Abstract

Background: Increasing bodies of epidemiological evidence indicate potential associations between dysphagia and the risk of frailty in older adults. We hypothesized that older adults with symptoms of dysphagia might have a higher prevalence of frailty or prefrailty than those without dysphagia. Methods: We systematically searched the PubMed, Embase, and Cochrane Library databases for relevant studies published through 20 April 2022. Cross-sectional and longitudinal studies that examined the associations between dysphagia and the existence of frailty or prefrailty in community-dwelling, facility-dwelling, or hospitalized adults aged 50 years or older were synthesized. The Newcastle–Ottawa Scale was used to evaluate study quality. Results: The meta-analysis comprised 12 cohorts, including 5,503,543 non-frailty participants and 735,303 cases of frailty or prefrailty. Random-effect meta-analysis demonstrated a significant association between dysphagia and the risk of frailty and prefrailty (OR, 3.24; 95% CI, 2.51–4.20). In addition, we observed consistent results across the subgroups and heterogeneity assessments. Conclusions: We propose including dysphagia assessment as a critical factor in the cumulative deficit model for identifying frailty in older adults. Understanding dysphagia and the potential role of nutritional supplements in older adults may lead to improved strategies for preventing, delaying, or mitigating frailty.

## 1. Introduction

Safe and effective swallowing is a bodily function essential to human life. Safe swallowing involves an alternating on-and-off interaction between respiration and swallowing and an integrated cough reflex [1,2]. Effective swallowing also requires intact muscular and neurocognitive coordination. However, these life-sustaining functions decline with advancing age [3]. Strategies for adapting to these hazardous aging-related changes, including modifications to food consistency, adoption of swallow postures and maneuvers, and cortical compensation [4], may help minimize the effect of such changes on quality of life; nevertheless, the accumulation of precipitating factors (e.g., decreased saliva production; worsening dental problems; reduced oral, pharyngeal, and esophageal mucosal sensitivity; and loss of muscle mass and strength) may lead to decompensation that engenders an increased susceptibility to swallowing dysfunction [5]. Consequently, swallowing difficulties manifest with aging [6,7].

Dysphagia is a subjective feeling of difficulty or discomfort in safely and effectively moving a dietary bolus from the oral cavity to the stomach. The difficulty or discomfort may involve the passage of the bolus from the oral cavity to the esophagus (i.e., oropharyngeal dysphagia), passage of the bolus from the esophagus to the stomach (i.e., esophageal dysphagia), or both. The prevalence of dysphagia increases with advanced age [8]. Using submental surface electromyography and nasal airflow measurement, Wang et al. [9] demonstrated that compared with young- and middle-aged participants, healthy older community-dwelling participants had a significantly delayed onset of swallowing, a longer duration of swallowing apnea, and a greater probability of piecemeal deglutition. In individuals aged younger than 60 years, dysphagia is usually associated with oncologic [10] and neurologic pathologies, but in older individuals, it is related to the aging process alone or engendered by neurological and neurodegenerative comorbidities [8,11]. Dysphagia is highly prevalent in different cohorts of older people [12], including community-dwelling individuals (11.4−33.7%), facility-dwelling individuals (38−51%), hospitalized individuals (29.4−47%), and hospitalized individuals with community-acquired pneumonia (55−91.7%), compared with the general population. Because dysphagia is a multifactorial disorder that is caused by multiple etiological factors, the relationship between dysphagia and the aging process or geriatric syndromes such as frailty, regardless of other comorbidities, remains a focus of research [12].

Frailty is characterized by a declining physiological reserve and loss of resistance to minor internal or external stressors caused by cumulative age-related functional deficits [13]. Fried et al. [14] described five elements of a frailty phenotype: low strength, slow motor performance, exhaustion, low physical activity, and recent unintentional weight loss. Dysphagia and frailty share the characteristics of a geriatric syndrome because they are highly prevalent among older adults [15,16]; are caused by multiple factors; are associated with several comorbidities [17]; predict poor clinical outcomes such as falls [18], disability [19], hospitalization [20,21], long-term care institutionalization, and mortality [16,22], and require a multidisciplinary approach for their improvement [11]. Timely nutritional intervention and rehabilitation in these patients could improve their survival and quality of life [23,24,25,26]. Therefore, recognizing amendable factors associated with the development of frailty is a crucial aspect of aging care [27,28,29,30].

Bahat et al. [5] reported an independent association of dysphagia with frailty scores—regardless of age, existence of neurodegenerative disorders, number of chronic diseases, or polypharmacy—in older adults selected from a geriatric outpatient clinic. Researchers have reported a strong association between deteriorated swallowing function and frailty [22,31], and an increasing number of studies have suggested that dysphagia is a risk factor for frailty in older adults [5,22,24,31,32,33,34,35]; nevertheless, the findings of epidemiologic research on the connections between dysphagia and frailty have been inconclusive [36,37,38,39]. Integrated studies investigating the association between dysphagia and frailty are inadequate. Moreover, whether dysphagia disorders in older people living in different settings, which could have multiple etiologies, are risk factors for frailty is unclear. Accordingly, we conducted a systematic review and meta-analysis of cross-sectional and longitudinal studies to substantiate the potential associations between dysphagia and frailty. We hypothesized that older adults with dysphagia might demonstrate an increased risk of frailty-related phenomena, regardless of phenotype, compared with those without dysphagia. Clarifying these associations might provide new perspectives for delaying the development of frailty in older adults.

## 2. Materials and Methods

### 2.1. Data Sources and Searches

To compare the occurrence of frailty and prefrailty in older adults with and without dysphagia, we systematically searched for articles published on PubMed, Embase, and the Cochrane Database of Systematic Reviews from their inception through 20 April 2022, without language restrictions. In addition, we reviewed the reference lists of the relevant articles to identify additional studies that were not found in the initial database search. The main search terms included “dysphagia,” “deglutition,” “dysfunction,” “older adults,” and “frailty.” To ensure a comprehensive search, we considered word variations for each of the senses, and medical terms. We did not identify any unpublished abstracts or conference proceedings that fulfilled our inclusion criteria. Our detailed search strategy can be found in the in the Methods S1 in the Supplementary Materials. We followed all the reporting standards listed in the Meta-Analysis of Observational Studies in Epidemiology checklist [40] (Methods S2 in the Supplementary Materials) and the Preferred Reporting Items for Systematic Review and Meta-Analyses (PRISMA) reporting guidelines. The Cardinal Tien Hospital Institutional Review Board waived the need for ethics approval for the pooled analysis.

### 2.2. Study Selection and Data Extraction

We included observational studies that examined associations between dysphagia and the risk of frailty among adults aged ≥50 years, including community-dwelling, facility-dwelling, and hospitalized older adults. Studies that exclusively reported selected risk groups and subpopulations with morbidities (e.g., stroke, brain tumors, head and neck cancers, esophageal cancer, or other gastrointestinal disorders that cause impaired swallowing function, irradiations, depression, dementia, or other psychiatric disorders) were excluded. This study has been registered in PROSPERO (CRD42022296590). We included the study protocol of the synthesis in Protocol in the Supplementary Materials.

The primary exposure of interest was impaired swallowing function, defined using a clinical diagnosis of dysphagia, objective or validated subjective assessments, and self-report questionnaires. An *International Classification of Diseases, Ninth Revision, Clinical Modification* (ICD-9-CM) diagnosis code for dysphagia, a positive result on the Eating Assessment Tool-10 (EAT-10) and other self-report questionnaires, and a failed or an abnormal water swallowing test indicated dysphagia. The outcome of the systematic review was the existence of frailty or prefrailty. In the Fried frailty phenotype assessment [14], non-frailty (or robust), prefrailty, and frailty are defined as syndromes that meet none, 1 or 2, and ≥3 of the following 5 criteria: an unintentional weight loss of 10 lbs in the past year, self-reported exhaustion, weakness of grip strength, slow walking speed, and low physical activity. The original [14] and modified [41,42,43,44] Fried frailty index (FFIs) were used to define frailty and prefrailty in our synthesis. Additionally, associations for different dysphagia and frailty assessment tools were included in a subgroup analysis.

Two investigators (A.-Y.Y. and R.-Y.Y.) independently abstracted baseline and outcome data. Any discrepancies were evaluated by J.-W.C. and discussed among all 3 reviewers. We extracted the following information from each study: study design, study population characteristics, namely age (mean with range), sex composition, geographic location, dysphagia assessment methods, frailty and prefrailty ascertainment, and adjusted covariates in statistical models. We contacted the corresponding authors of eligible studies whenever we needed to obtain additional information that was not available in the article or online supplementary files. Thus, the information was extracted directly from the included studies or provided by the corresponding authors. Pooled odds ratios (ORs) were calculated from the crude number of participants stratified by dysphagia and frailty development.

### 2.3. Risk-of Bias Assessment

Two authors (Y.-C.C. and J.-W.C.) independently appraised the methodological quality of each study by using the Newcastle–Ottawa scale (NOS) [45]. The Cochrane Collaboration has acknowledged the NOS for evaluating the risk of bias at the outcome level [46]. For longitudinal studies, the NOS assigns a score out of 9 to each study (where 9 indicates that the study meets all 9 criteria for quality assessments and 0 indicates that the study does not meet any of the criteria) on the basis of potential domains of bias, such as selection, comparability, and outcome. For cross-sectional studies, the modified NOS [47] assigns a score out of 10 to each study. Quality assessment data individually appraised by each of the reviewers were compared. If consensus could not be achieved, Y.-C.C., J.-W.C., and S.-D.L. discussed the discrepancies for adjudication. Quality scores of <5 were considered to indicate a high risk of bias, whereas scores of 5 to 7 and ≥8 were considered to indicate a moderate or low risk of bias [48]. Studies evaluated as having a high risk of bias were excluded in subsequent sensitivity analysis.

### 2.4. Data Synthesis and Analysis

We recorded and analyzed the study data between September 2021 and January 2022 by using Review Manager (RevMan), Version 5.4 (The Cochrane Collaboration, London, United Kingdom). We calculated pooled ORs by conducting a random-effects meta-analysis of raw data extracted from each study. The precision levels of the effect sizes are expressed as 95% CIs. We assessed the statistical heterogeneity and inconsistency of the effects across the included studies by using the Cochran Q test (*p* < 0.10) and *I*^2^ statistics, respectively. Studies with a high risk of bias, with different study designs, or with significant heterogeneity were excluded from subsequent subgroup analyses. We performed the subgroup analyses by stratifying the data by age, residence, sample size, assessment tools, and geographic region to characterize potential sources of heterogeneity. In our subgroup analyses, we calculated the risk estimates for outcomes of frailty alone or frailty and prefrailty. Additionally, we conducted sensitivity analyses by sequentially excluding individual studies to examine the effect of such exclusions on the overall risk estimate. Publication bias was evaluated visually through funnel plots. A 2-sided *p* value of <0.05 was deemed statistically significant.

## 3. Results

### 3.1. Study Selection and Baseline Characteristics

**Figure 1:**
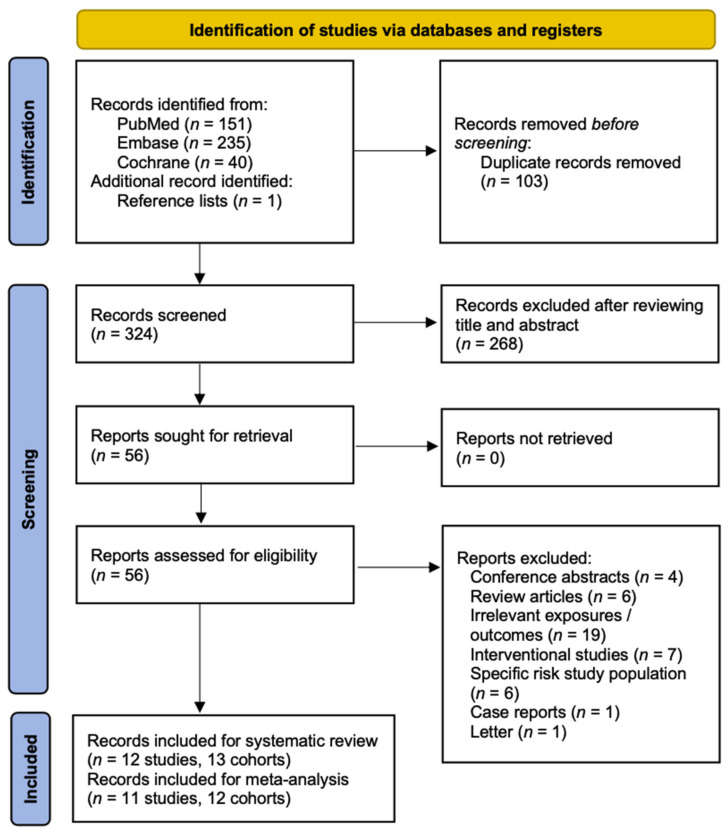
Flow Diagram of the Study Selection Process.

We searched 151, 235, and 40 records from the PubMed, Embase, and Cochrane (Methods S1 in the Supplementary Materials) databases, respectively. A search of the reference lists yielded one additional article. We screened a total of 427 studies. After screening for duplicates and the titles and abstracts of the studies, we excluded 371 studies; thus, we reviewed the full texts of the remaining 56 studies. We included a total of 12 studies in our systematic review, 11 of which were quantitatively synthesized. Among them, one study [36] compared two cohorts from England and Japan, and we meta-analyzed these cohorts separately. Figure 1 illustrates a flow diagram of the literature search and screening results.

Table 1 presents a summary of the 12 studies (13 cohorts); of these studies, 11 (12 cohorts) used cross-sectional data [5,22,24,31,32,33,34,35,36,37,38] and one used longitudinal data [39] to assess the association between dysphagia and frailty. Of the 13 cohorts, ten comprised community-dwelling older adults (*n* = 9817) [5,22,24,31,34,36,37,38,39], two comprised hospitalized patients (*n* = 6,230,500) [33,35], and one comprised facility-dwelling older adults (*n* = 592) [32]. Regarding geographic location, among the studies, six were conducted in Japan [22,24,31,34,36,39], two were conducted in the United States [33,38], and the remaining five were conducted in Turkey [5], Australia [32], England [36], Taiwan [37], and China [35] separately.

Most of the cohorts (8/13, 61.5%) used the FFI to define frailty [5,24,35,36,37,38,39]; the remaining cohorts defined frailty by using the Frailty Index (1/13, 7.7%) [22], a modified 36-item electronic Frailty Index (1/13, 7.7%) [32], the 10-item Johns Hopkins Adjusted Clinical Groups Frailty Risk Score (1/13, 7.7%) [33], and the 25-item Kihon Checklist (2/13, 15.4%) [31,34]. Among the 13 cohorts, eight reported a prevalence of both frailty and prefrailty in the corresponding cohorts [5,24,31,32,33,35,37,38], but only four of them [31,35,37,38] demonstrated data related to the association between dysphagia and frailty or prefrailty. To determine the development of dysphagia, unstructured self-report questionnaires were administered to six cohorts [32,34,36,37,39], validated questionnaires were administered to four cohorts (the 10-item Eating Assessment Tool administered to three cohorts [5,24,31] and the Dysphagia Severity Scale administered to one cohort [22]), ICD-9-CM codes on discharge records were used in one cohort [33], a water swallowing test was applied to one cohort [35], and both unstructured self-report questionnaires and a water swallowing test were administered to one cohort [38].

All 12 studies (13 cohorts) reported an association between dysphagia and the risk of frailty. Statistical adjustments varied across the studies (Table 1). The included studies provided data on the numbers of participants with dysphagia and frailty or prefrailty; alternatively, the corresponding authors provided the necessary data upon request. Using these data, we calculated cumulative ORs. One cross-sectional study [5] did not report the exact case counts, and we could not obtain data on these counts after contacting the corresponding authors. Therefore, we excluded this study from our quantitative synthesis. Finally, the meta-analysis included 11 studies (12 cohorts) with 5,503,543 participants with non-frailty and 735,303 participants with frailty or prefrailty.

### 3.2. Studies with Cross-Sectional Data

Cross-sectional data on the association between dysphagia and frailty were available in 12 cohorts (a total of 6,238,898 participants). The sample size ranged from 47 in one of the cohorts [38] to 6,230,114 [33]. A total of one cohort [38] included only female participants, but the remaining included mixed-gender cohorts (ranging from a 42.7% female representation [22] to an 83.8% female representation [24]). The mean age of the cohorts ranged from 70.1 years [33] to 87 years [36]. There were two studies reported with a median age that ranged from 72.9 years [31] to 88 years [32].

### 3.3. Dysphagia and Incident Frailty in One Longitudinal Study

A total of 2011 community-dwelling older adults in one longitudinal cohort [39] conducted in Japan were enrolled to examine the presence of poor oral health and the subsequent development of frailty. Of these older adults, 50.6% were women; the mean age of the study population was 73 years. The follow-up period was 4 years. This longitudinal study revealed that participants who presented with poor oral health at baseline demonstrated a 2.4-fold increased risk of physical frailty compared with those without poor oral health; a possible explanation for this finding is that poor oral health causes subjective eating and swallowing difficulties. This longitudinal study provided data on the number of participants with symptoms of dysphagia at baseline and data on cases of incident physical frailty during follow-up; therefore, we incorporated these data into our meta-analysis.

### 3.4. Meta-Analysis of Dysphagia and Frailty

**Figure 2:**
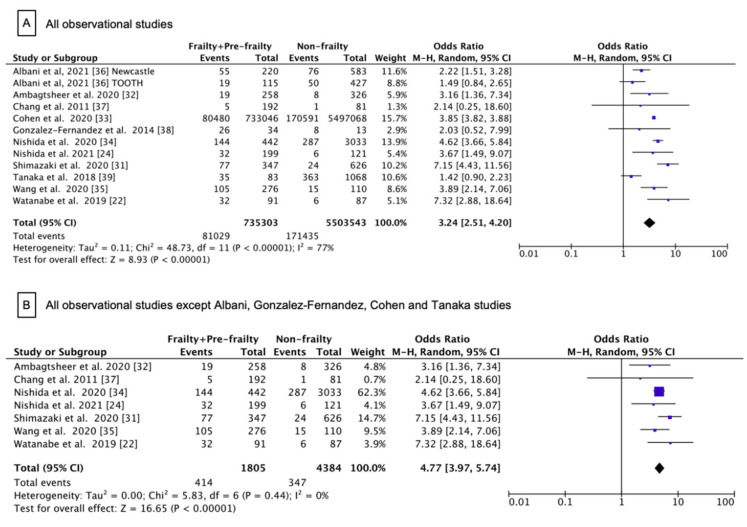
Random-effects meta-analysis results of the association between dysphagia and the risks of frailty and prefrailty. (**A**) Results obtained when all observational studies were included in the meta-analysis. (**B**) Results obtained when all observational cohorts except for those of Albani, Cohen, Gonzalez–Fernandez, and Tanaka were included in the meta-analysis [22,24,32,33,34,35,36,37,38,39].

A random-effect meta-analysis of the 12 observational cohorts revealed a significant association between dysphagia and the risk of frailty or prefrailty (pooled OR = 3.24; 95% CI = 2.51–4.20; *p* < 0.00001; Figure 2A). We noted a high degree of heterogeneity between the studies (*I*^2^ = 77%, *p* < 0.00001). Exploring the source of heterogeneity revealed that when we excluded studies with relatively low methodological quality [38], different study designs [39], a considerably older study population [36], and extremely large sample sizes [33], the estimated risk effect increased (pooled OR = 4.77; 95% CI = 3.97–5.74; *p* < 0.00001; Figure 2B), and no heterogeneity was observed (*I*^2^ = 0%; *p* = 0.44). Moreover, no significant asymmetry was observed, as displayed in the funnel plots presented in Figure S1 in the Supplementary Materials.

### 3.5. Subgroup Meta-Analysis

Table 2 presents a summary of the results obtained from our subgroup meta-analysis in which we stratified the studies by their characteristics. To minimize heterogeneity, we included seven cohorts [22,24,31,32,34,35,37] in the subgroup meta-analysis. Additionally, we separately derived the main estimates for the odds of frailty alone and those of frailty and prefrailty. The studies were stratified according to age (mean age of study participants: >80 vs. 70≤ age <80 years), residence (community- vs. facility-dwelling vs. hospitalized), sample size (<500 vs. ≥500), assessment tools used (for frailty: FFI vs non-FFI; for dysphagia: self-report questionnaires vs. EAT-10 or Dysphagia Severity Scale vs. water swallow test), and geographic locations (United States and Australia vs. Asia). We observed large effect estimates for the cross-sectional associations between dysphagia and the risk of frailty alone and the risk of frailty and prefrailty across all subgroups. We determined no significant differences between the effect estimates for age, residence, sample size, assessment tools, and geographic region. The pooled ORs for the association between dysphagia and the risk of frailty alone were uniformly higher than those for the association between dysphagia and the risk of frailty and prefrailty, except in the younger age subgroup (70 ≤ age < 80 years). A relatively high degree of heterogeneity (*I*^2^ ≥ 50%) was observed in the subgroups of studies with relatively large sample sizes (≥500, *I*^2^ = 69%, *p* = 0.04) and studies that used non-FFI tools to assess the development of frailty (*I*^2^ = 57%, *p* = 0.07). However, in subgroup analysis of the association between dysphagia and the risk of frailty and prefrailty, the degree of heterogeneity decreased (*I*^2^ = 46%, *p* = 0.61).

Subsequently, we performed a subgroup analysis according to age, sample size, assessment tools, and geographic location; this analysis targeted the five cohorts that comprised only community-dwelling participants (Table 2). The pooled ORs for this congeneric study population remained unchanged. Similarly, a high degree of heterogeneity was observed in the subgroups of cohorts with relatively large sample sizes and studies that used non-FFI tools to assess the development of frailty (*I*^2^ = 81%, *p* = 0.02 and *I*^2^ = 65%, *p* = 0.06, respectively). The pooled ORs for the association between dysphagia and the risk of frailty were also higher than those for the association between dysphagia and the risk of frailty and prefrailty, except in the subgroup of studies that implemented self-report questionnaires for assessment.

### 3.6. Assessment of Methodological Quality and Publication Bias

Table S1 in the Supplementary Materials presents the results of critical appraisal for the individual cohorts. Of the eleven cross-sectional studies, ten attained an NOS score of 5 or higher (moderate to low risk of bias). We deemed one study to have a relatively high risk of bias (NOS score: 4) because of its unrepresentative study population (comprising only female participants), small and unjustified sample size, and lack of statistical adjustments for confounding factors. However, excluding this study did not alter our risk estimate (Table S2 in the Supplementary Materials). The included longitudinal study had an NOS score of 8 out of 9. A visual inspection of the funnel plot did not reveal publication bias (Figure S1 in the Supplementary Materials).

## 4. Discussion

This systematic review and meta-analysis of cross-sectional studies revealed that the presence of dysphagia was significantly associated with greater odds of frailty. Evidence from one longitudinal study also suggested that dysphagia increases the risk of frailty. These findings were consistent across subgroups defined by age, residence, sample size, assessment tools used, and geographic locations and remained robust in sensitivity analyses. Exploratory analyses of heterogeneity modestly strengthened this association. Moreover, the calculated ORs for the association between dysphagia and frailty alone were generally higher than those observed for the association between dysphagia and frailty and prefrailty. According to our review of the literature, the current study provides the most comprehensive evidence of dysphagia being a potential risk factor for the development and progression of frailty.

Aging is an ongoing global health concern. The total number of people aged ≥60 years worldwide is estimated to rise from approximately one billion in 2020 to two billion by 2050 [49]. Notably, according to experts, the older-age dependency ratio, defined as the number of people aged ≥65 years per 100 working-age adults (aged 20–64 years), is projected to nearly double from 17 in 2020 to 30 in 2050; this signifies that the ratio of working-age adults to older persons will drop to only 3:1 [50]. To address this trend, the World Health Organization initiated an action plan in 2020 to improve functional ability, intrinsic capacity, and environment, which are considered crucial components for achieving the goal of “healthy aging” [51]. Of these components, optimizing functional ability in older adults is the most effective for achieving independent and healthy aging.

In clinical geriatric medicine, frailty typically refers to progressive multisystem dysfunction and increasing vulnerability. The American version of the *International Classification of Diseases*, *Tenth Revision, Clinical Modification* [52] includes the diagnostic code R54 for reimbursement claims effective from 1 October 2020; this code is currently applicable for defining clinical entities not otherwise specified, including frailty, old age, senescence, senile asthenia, and senile debility, except for age-related cognitive decline, sarcopenia, senile psychosis, and senility. Because of the lack of a rigid set of aging biomarkers and the variance in the onset time of the transition from dysfunction to disease among elderly people, controversy remains about whether aging represents a disease that should be included in the upcoming *International Classification of Diseases, Eleventh Revision, Clinical Modification* diagnosis codes [53].

Several pathways may underpin the observed association between dysphagia and frailty [28]. Older adults with dysphagia are more prone to having nutritional deficiencies [32] and are thus expected to experience frailty more commonly than do those without dysphagia [5,22,24,54]. A considerable proportion of our observed association between dysphagia and risk of frailty can be attributed to undernutrition [17,28]. Undernourishment has been considered to be a core component of the frailty cycle [55]. Chronic undernutrition may cause a vicious cycle of weight loss, sarcopenia, decreased energy expenditure, and reduced metabolic rate, leading to multiple phenotypes of frailty, such as low activity, slow walking speed, low grip strength, and exhaustion [55]. Additionally, undernutrition in older adults can have multiple adverse effects, including falls [56], repeated infections, depression, and sarcopenia [57]. Notably, these poor outcomes were also reported to be correlated with the existence of frailty [16]. The complex relationship between dysphagia, malnutrition, and frailty was recently clarified by Nishida [24], who reported that in community-dwelling older adults, dysphagia was independently associated with either nutritional status or frailty. Because community-dwelling older adults receive nutrition orally [58,59], dysphagia may cause malnutrition and subsequent weight loss, thereby predisposing them to frailty [60]. Early screening, multicomponent intervention [56,61], and interdisciplinary management [62] may improve outcomes in this population.

Compared with community-dwelling older adults, those living in long-term care units, facilities, or hospitals may have a greater chance of receiving tube feeding [63]. For older adults who receive such feeding, undernutrition is a less likely mediator in the relationship between dysphagia and a greater risk of frailty. Additional factors may influence our observed association between dysphagia and frailty, including depressed mood [32,34,37], masticatory sarcopenia [39], polypharmacy [22,37], or the presence of multiple comorbidities [12,54]. However, initial symptoms of dysphagia are often overlooked because their onset is slow and inconspicuous; such symptoms manifest in patients only upon repeated hospitalization due to community-acquired pneumonia (CAP) [11]. Notably, the observed prevalence of dysphagia in older patients hospitalized with CAP was as high as 91.7% [64]. Evidence also reveals that the cause of pneumonia was aspirational rather than inhalational [65]. Accordingly, repeated hospital admission and polypharmacy may further predispose older adults to frailty [19,20]. Observational studies also demonstrated that individuals with frailty and dysphagia have an increased risk of aspiration pneumonia and mortality [15,66], regardless of functional status or comorbidities. Older adults with frailty who were bedridden or did not survive were thus censored from the study populations examined to determine the association between dysphagia and the risk of frailty [38]. Therefore, survival bias may have caused an underestimation of the true association between dysphagia and the risk of frailty. The actual association between dysphagia and frailty may have been stronger than we observed. Currently, an increasing proportion of healthy people are completing advanced directives to avoid long-term artificial nutrition during their end-of-life stages [67,68]; therefore, the early detection of swallowing dysfunction and an effective intervention will become increasingly essential.

The majority of the included cohorts involved older adults in different settings who did not have diseases that increased their susceptibility to dysphagia, such as neurodegenerative disease and oropharyngeal organic disease, and they did not receive gastric tube or tracheal intubation [35]. However, the included cohorts used different assessment tools for identifying dysphagia and frailty, and this may be a source of heterogeneity between the cohorts. Initially, we observed a high degree of heterogeneity (*I*^2^ = 77%) between the cohorts identified from the search. Sensitivity analysis (Table S2 in the Supplementary Materials) demonstrated that the exclusion of the results of four cohorts [33,36,39] caused the heterogeneity to decrease significantly. Nevertheless, Gonzalez–Fernandez et al. [38] included only a population of oldest–old women; in the quality assessment, we observed a high risk of bias for this study. Therefore, we included only seven cohorts in our subsequent subgroup analyses. Overall, the link between dysphagia and frailty and prefrailty remained consistent in the sensitivity analysis. A notable finding in our subgroup analyses is that for older people aged between 70 and 80 years, the odds of frailty alone (4.69; 95% CI, 2.06–10.69) were smaller than those of any frailty (4.80; 95% CI, 3.92–5.87), and this may be due to the potential heterogeneity between the five included cohorts. When we examined only the results from four cohorts that included community-dwelling older adults, the odds of frailty in adults aged 70–80 years were greater than those of any frailty (5.66; 95% CI, 3.51–9.12 vs. 5.01; 95% CI, 3.80–6.59). Further exploration of heterogeneity revealed that the excluded study [35] with hospitalized participants adopted 30-mL water swallow tests to assess the development of dysphagia, which differed from the self-report questionnaires used in the other four cohorts. Therefore, methodological differences may explain the observed discrepancy. Similarly, in subgroup analyses of two cohorts that included community-dwelling participants and used self-report questionnaires for dysphagia identification, the odds of frailty (4.53; 95% CI, 3.62–5.66) were smaller than those of any frailty (4.58; 95% CI, 3.63–5.78). However, instead of a standalone questionnaire, one [34] of these meta-analyzed cohorts used oral function on the frailty checklist to determine the presence of dysphagia. We presumed that the discordant results of the subgroup analyses may be due to the small number of included cohorts and the various analytical methods used.

Overall, optimized functional abilities and intrinsic capacities as well as a friendly environment are crucial to achieving the goal of healthy aging. On the basis of our meta-analysis results, we propose that safe and effective swallowing is an essential component to sustaining healthy aging (Figure S2 in the Supplementary Materials). This is because when adequately preserved, each life-supporting and psychological function can protect older adults from developing frailty and help them enjoy a sustainable, healthy life as they age.

### Strengths and Limitations

The strengths of this meta-analysis include the comprehensive synthesis of all available observational studies with large sample sizes, findings across several analytic methods and different subgroups, and assessment of a potential dose–response association. Nonetheless, our study has several limitations. First, five of the twelve cohorts included in our meta-analysis did not use the original Fried criteria for frailty assessment, which may have influenced the quality of the synthesis. Because these five cohorts used validated assessment tools for frailty identification, we combined their corresponding results and performed subgroup analyses. Our findings remain consistent regardless of the frailty criteria used. Similarly, the included cohorts adopted different tools for defining dysphagia; five used simple self-administrated questionnaires, four used validated questionnaires, two used objective water swallow tests, and one used *ICD-9-CM* codes. Nevertheless, our subgroup analyses still yielded a positive association between dysphagia and frailty risk. Second, most of the included cohorts relied on self-report tools for identifying dysphagia and frailty, which may involve inherent measurement errors and misclassifications [41]. However, eleven of the twelve included cohorts were determined to have a low to moderate risk of bias in our quality assessment [43]. Third, we collected unadjusted baseline and outcome data in the meta-analysis, which may have introduced bias from potential confounding factors. However, all associations remained robust in the covariate-adjusted subgroups, suggesting that no individual subgroup alone affected the pooled estimates. Nevertheless, we note that some subgroups had a limited number of cohorts for detecting associations, and the corresponding findings should be interpreted with caution. Furthermore, only four cohorts [34,38,40,41] reported separate data regarding frailty and prefrailty; thus, we did not perform subsequent subgroup analyses for the individual risk of prefrailty specifically. The pooled ORs for frailty were mainly higher than those for frailty and prefrailty combined, suggesting a potential dose–response effect; however, additional studies examining dysphagia and the corresponding risk of frailty and prefrailty are warranted to confirm this dose–response effect. Fourth, we included the Cohen study in the synthesis, which enrolled hospitalized patients aged 50 or more and a large sample size. The younger study population differed from the current definition of older people aged 65 years and above. The large sample size might affect the pooled estimates in the meta-analysis. However, sensitivity analysis without the Cohen study yielded increased pooled ORs. Therefore, the inclusion of relatively young participants could be considered a plausible confounder in the meta-analysis for generalizability. In addition, evidence from longitudinal data is scarce; causal relationships or pathway directions could not be inferred in cross-sectional studies. Moreover, the limited number assessed databases might not include all existing literature in multiple languages. Finally, most of the currently available studies have been conducted in East Asia. Future longitudinal studies including populations of various ethnicities outside East Asia are warranted for generalizability and to identify the causal relationship between dysphagia and the risk of frailty.

## 5. Conclusions

This study demonstrated that older adults who report signs and symptoms of swallowing dysfunction are at an increased risk of frailty or prefrailty. We recommended routine screening for dysphagia through subjective or objective methods in comprehensive geriatric assessments. Future longitudinal studies controlling for possible confounders, particularly nutritional status, depression, sarcopenia, polypharmacy, and comorbidities, are warranted to understand the causal relationship between dysphagia and frailty. Our review results indicate the need for frailty prevention programs to screen older adults for swallowing dysfunction and implement timely interventions. A more precise understanding of the role of dysphagia and potential benefits from nutritional supplements in the aging process may lead to improved strategies for preventing, delaying, or mitigating frailty.

## Figures and Tables

**Table 1:** Baseline Characteristics of Published Studies Included in the Systematic Review.

Source	Country	Participants No. and Dwelling	Study Design	Age, Mean Range, y	Female, *N* (%)	Dysphagia Ascertainment	Frailty Ascertainment	Baseline Prevalence of Frailty and Pre-Frailty *N* (%)	Adjustment for Original Investigation
Chang et al. [37], 2011	Taiwan	275 Community-Dwelling	Cross-sectional	71.1 65–79	148 (53.8)	Self-reported	FFI	Pre-frailty 161 (58.5) Frailty 31 (11.3)	Age, education status, history of falls in 1 year, pain history, depression, polypharmacy, timed up and go, number of comorbidities, MMSE score, and Barthel Index score
Gonzalez-Fernandez et al. [38], 2014	United States	47 Community-Dwelling	Cross-sectional	86.3 85–94	47 (100)	Self-reported and 3-Ounce water swallowing test	FFI	Pre-frailty 28 (59.6) Frailty 6 (12.7)	NA
Tanaka et al. [39], 2018 (Kashiwa Study)	Japan	2011 Community-Dwelling	Longitudinal	73.0 ≥65	1017 (50.6)	Self-reported	FFI	Frailty 1151 (57.2)	Age, sex, BMI, chronic conditions, cognitive function, depressive symptoms, living arrangements, yearly income, and current smoking status
Bahat et al. [5], 2019	Turkey	1138 Community-Dwelling	Cross-sectional	74.1 ≥60	790 (69.4)	EAT-10	FRAIL scale	Pre-frailty 514 (45.3) Frailty 325 (28.6)	Age, sex, presence of neurodegenerative diseases, number of chronic diseases and drugs, HGS (handgrip strength), UGS (usual gait speed), and nutritional status
Watanabe et al. [22], 2019 (ONEHOME)	Japan	178 Community-Dwelling	Cross-sectional	80.2 66–90	76 (42.7)	DSS	Frailty Index	Frailty 91 (51.1)	Age and sex
Ambagtsheer et al. [32], 2020	Australia	592 Facility-Dwelling	Cross-sectional	88.0 (median) ≥75	394 (66.6)	Self-reported	Modified 36-Item eFI	Pre-frailty 274 (46.3) Frailty 258 (43.6)	Model 1: age, sex, and facility characteristics (size, rurality) Model 2: Model 1 + 12 ACFI domains Model 3: Model 2 + five most prevalent conditions (arthritis, diabetes, hypertension, osteoporosis, and vision problems)
Cohen et al. [33], 2020 (NIS-HCUP-AHRQ)	United States	6,230,114 Hospitalized	Cross-sectional	70.1 ≥50	3,264,580 (52.4)	ICD-9-CM codes	ACG and FRS	Pre-frailty 1,295,864 (20.8) Frailty 43,611 (0.7)	Age, sex, race, hospital characteristics, geographic region, insurance, smoking status, household income, and admission type
Nishida et al. [34], 2020	Japan	3475 Community-Dwelling	Cross-sectional	75.8 ≥65	1920 (55.3)	Self-reported	25-Item Kihon Checklist	Frailty 419 (12.1)	Age, sex, domains of oral function, nutrition, physical function, homebound status, cognitive function, and depressive mood
Shimazaki et al. [31], 2020	Japan	978 Community-Dwelling	Cross-sectional	M: 73.2 F: 72.9 (median) 65–85	510 (52.1)	EAT-10	25-Item Kihon Checklist	Pre-frailty 295 (30.3) Frailty 81 (8.3)	Age, sex, BMI, hypertension, and stroke
Wang et al. [35], 2020	China	386 Hospitalized	Cross-sectional	74.8 65–93	190 (49.2)	30-mL water swallow test	FFI	Pre-frailty 182 (47.2) Frailty 94 (24.4)	Sex, number of chronic diseases, and history of choking/coughing while drinking
Nishida et al. [24], 2021	Japan	320 Community-Dwelling	Cross-sectional	77.3 ≥65	268 (83.8)	EAT-10	FFI	Pre-frailty 154 (48.1) Frailty 45 (14.1)	Age, sex, family structure, and self-rated health
Albani et al. [36], 2021 (Newcastle 85+ Study)	England	853 Community-Dwelling	Cross-sectional	85.0 >85	530 (62.1)	Self-reported	FFI	Pre-frailty 433 (53.9) Frailty 226 (28.1)	Age, sex, BMI, alcohol intake, smoking status, social class, cardiovascular disease, diabetes, hypertension, neuropsychiatric disease, and other health conditions
Albani et al. [36], 2021 (TOOTH Study)	Japan	542 Community-Dwelling	Cross-sectional	87.0 >85	306 (56.5)	Self-reported	FFI	Pre-frailty 339 (62.5) Frailty 120 (22.1)	Age, sex, BMI, alcohol intake, smoking status, social class, cardiovascular disease, diabetes, hypertension, neuropsychiatric disease, and other health conditions

Abbreviations: ACFI: Australian Aged Care Funding Instrument; ACG: 10-item Johns Hopkins Adjusted Clinical Groups; DSS: Dysphagia Severity Scale; EAT-10: 10-item Eating Assessment Tool; eFI: Electronic Frailty Index; FFI: Fried Frailty Index; FRS: Frailty Risk Score; NA: not applicable; NIS-HCUP-AHRQ: The National Inpatient Sample for the Healthcare Cost and Utilization Project by the Agency for Healthcare Research and Quality; NOS: Newcastle–Ottawa Scale; ONEHOME: The Observational Study of Nagoya Elderly with Home Medical study; TOOTH: The Tokyo Oldest Old Survey on Total Health.

**Table 2:** Subgroup Analysis of All Cross-Sectional Studies for Associations Between Dysphagia and Risk of Frailty.

Characteristic	Odds of Frailty				Odds of Frailty and Pre-Frailty	
	Studies, No.	Pooled Odds Ratio [95% CI]	I ^2^, %	*p* Value for Subgroup Differences	Studies, No.	Pooled Odds Ratio [95% CI]
Main estimate	7	5.22 [3.96, 6.89]	22%		7	4.77 [3.97, 5.74]
Study population				0.44		
Community-dwelling	5	5.79 [3.91, 8.58]	38%		5	5.08 [4.04, 6.39]
Hospitalized	1	5.11 [2.58, 10.13]	-		1	3.89 [2.14, 7.06]
Facility-dwelling	1	3.16 [1.36, 7.34]	-		1	3.16 [1.36, 7.34]
Geographic location				0.22		
US/Australia	1	3.16 [1.36, 7.34]	-		1	3.16 [1.36, 7.34]
Asia	6	5.50 [4.10, 7.38]	23%		6	4.87 [4.04, 5.89]
Sample size				0.94		
*N* < 500	4	5.22 [3.29, 8.29]	0%		4	4.29 [2.79, 6.59]
*N* ≥ 500	3	5.36 [3.07, 9.38]	69%		3	5.01 [3.50, 7.18]
Mean age				0.75		
70 ≤ age < 80	5	4.69 [2.06, 10.69]	32%		5	4.80 [3.92, 5.87]
Age ≥ 80	2	5.22 [3.96, 6.89]	42%		2	4.69 [2.06, 10.69]
Assessment tools for frailty in all study populations				0.61		
FFI	3	5.63 [3.56, 8.89]	0%		3	3.71 [2.29, 6.03]
Non-FFI	4	5.22 [3.96, 6.89]	57%		4	5.20 [3.82, 7.07]
Assessment tools for dysphagia in all study populations				0.44		
Self-reported	3	4.53 [3.62, 5.66]	0%		3	4.46 [3.57, 5.58]
EAT-10 or DSS	3	6.86 [3.74, 12.60]	41%		3	6.36 [4.32, 9.36]
Water swallow test	1	5.11 [2.58, 10.13]	-		1	3.89 [2.14, 7.06]
Community-dwelling study populations	5	5.79 [3.91, 8.58]	38%		5	5.08 [4.04, 6.39]
Sample size						
*N* < 500	3	5.32 [2.85, 9.94]	0%	0.70	3	4.77 [2.56, 8.88]
*N* ≥ 500	2	6.42 [3.05, 13.51]	81%		2	5.46 [3.59, 8.32]
Mean age						
70 ≤ age < 80	4	5.66 [3.51, 9.12]	49%	0.63	4	5.01 [3.80, 6.59]
Age ≥ 80	1	7.32 [2.88, 18.64]	-		1	7.32 [2.88, 18.64]
Assessment tools for frailty in Community-dwelling study populations				0.37		
FFI	2	4.11 [1.77, 9.52]	0%		2	3.39 [1.47, 7.80]
Non-FFI	3	6.47 [3.74, 11.19]	65%		3	5.57 [3.97, 7.81]
Assessment tools for dysphagia in Community-dwelling study populations				0.21		
Self-reported	2	4.53 [3.62, 5.66]	0%		2	4.58 [3.63, 5.78]
EAT-10 or DSS	3	6.86 [3.74, 12.60]	41%		3	6.36 [4.32, 9.36]

## Data Availability

Not applicable.

## Supplementary Materials

The following are available online at https://www.mdpi.com/article/10.3390/nu14091812/s1, Supporting Information, including Table S1: Critical appraisal of the studies, Table S2: Sensitivity analysis, Figure S1: Funnel plots showing potential publication bias, Figure S2: Illustration of potential linkage between dysphagia and frailty, Methods S1: Detailed Search Strategy, Methods S2: MOOSE checklist, and Protocol, included ref. [69].
