# Supplementary material for: Association between Dysphagia and Frailty in Older Adults: A Systematic Review and Meta-Analysis
Source: Nutrients. 2022 Apr 27;14(9):1812. doi: 10.3390/nu14091812 (PMC9105461; doi:10.3390/nu14091812)
Supplement: Supplementary file 1 [file nutrients-14-01812-s001.zip › nutrients-1687883-supplementary.pdf]

**Supporting Information**

Title: Association between Dysphagia and Frailty in Older Adults: A Systematic Review and Meta-Analysis


| | Table of Contents | Page |
|---|---|---|
| 1 | Table S1: Critical appraisal of the studies | 2 |
| 2 | Table S2: Sensitivity analysis | 3 |
| 3 | Figure S1: Funnel plots showing potential publication bias | 4 |
| 4 | Figure S2: Illustration of potential linkage between dysphagia and frailty | 5 |
| 5 | Methods S1: Detailed Search Strategy | 6 |
| 6 | Methods S2: MOOSE checklist | 9 |
| 7 | Protocol | 11 |

**Table S1. Newcastle–Ottawa Scale for Critical Appraisal of Cross-Sectional and Longitudinal Studies**

| | | Selection | | | | Comparability | Outcome | | |
|---|---|---|---|---|---|---|---|---|---|
| **Source** | **Study design** | **Representativeness of the sample** | **Sample size** | **Non-respondents** | **Ascertainment of exposure** | **Based on design and analysis** | **Assessment of outcome** | **Statistical test** | **Total score** |
| Chang 2011 | Cross-sectional | +1 (b) | +0 (b) | +0 (c) | +1 (b) | +2 (a & b) | +2 (b) | +1 (a) | 7/10 |
| Gonzalez-Fernandez 2014 | Cross-sectional | +0 (c) | +0 (b) | +0 (c) | +2 (a) | +0 | +2 (a) | +0 (b) | 4/10 |
| Bahat 2019 | Cross-sectional | +0 (c) | +0 (b) | +0 (c) | +2 (a) | +2 (a & b) | +2 (b) | +1 (a) | 7/10 |
| Watanabe 2019 | Cross-sectional | +1 (b) | +0 (b) | +1 (a) | +2 (a) | +0 | +1 (c) | +0 (b) | 5/10 |
| Ambagtsheer 2020 | Cross-sectional | +1 (b) | +0 (b) | +0 (c) | +0 (c) | +2 (a & b) | +1 (c) | +1 (a) | 5/10 |
| Cohen 2020 | Cross-sectional | +1 (a) | +1 (a) | +1 (a) | +0 (c) | +2 (a & b) | +2 (b) | +1 (a) | 8/10 |
| Nishida 2020 | Cross-sectional | +1 (a) | +0 (b) | +0 (b) | +1 (b) | +2 (a & b) | +1 (c) | +1 (a) | 6/10 |
| Shimazaki 2020 | Cross-sectional | +1 (b) | +0 (b) | +0 (b) | +2 (a) | +2 (a & b) | +1 (c) | +1 (a) | 7/10 |
| Wang 2020 | Cross-sectional | +1 (b) | +0 (b) | +0 (c) | +2 (a) | +2 (a & b) | +2 (b) | +1 (a) | 8/10 |
| Nishida 2021 | Cross-sectional | +1 (b) | +0 (b) | +0 (c) | +2 (a) | +2 (a & b) | +1 (c) | +1 (a) | 7/10 |
| Albani 2021 (Newcastle 85+) | Cross-sectional | +1 (b) | +0 (b) | +0 (c) | +1 (b) | +2 (a & b) | +1 (c) | +1 (a) | 6/10 |
| Albani 2021 (TOOTH) | Cross-sectional | +1 (b) | +0 (b) | +0 (c) | +1 (b) | +2 (a & b) | +1 (c) | +1 (a) | 6/10 |

| | | Selection | | | | Comparability | Outcome | | | |
|---|---|---|---|---|---|---|---|---|---|---|
| **Source** | **Study design** | **Representativeness of the exposed cohort** | **Selection of the non-exposed cohort** | **Ascertainment of exposure** | **Outcome was not present at start** | **Based on design and analysis** | **Assessment of outcome** | **Follow up long enough** | **Adequacy of follow up** | **Total score** |
| Tanaka 2018 | Longitudinal | +1 (b) | +1 (a) | +1 (b) | +1 (a) | +2 (a & b) | +1 (b) | +0 (b) | +1 (b) | 8/9 |

**Table S2. Sensitivity Analysis**

| Sequential Exclusion of Studies | Odds of Frailty and Pre-Frailty | | |
|---|---|---|---|
| | Pooled Odds Ratio [95% CI] | $I^2$, % | *P* Value for Heterogeneity |
| All included studies | 3.24 [2.51, 4.20] | 77% | < .00001 |
| One study excluded | | | |
| Chang 2011 | 3.26 [2.51, 4.24] | 79% | < .00001 |
| Gonzalez-Fernandez 2014 | 3.29 [2.53, 4.28] | 79% | < .00001 |
| Tanaka 2018 (Kashiwa Study) | 3.61 [2.87, 4.55] | 67% | .0008 |
| Watanabe 2019 (ONEHOME) | 3.10 [2.38, 4.05] | 79% | < .00001 |
| Ambagtsheer 2020 | 3.25 [2.48, 4.26] | 79% | < .00001 |
| Cohen 2020 (NIS-HCUP-AHRQ) | 3.14 [2.14, 4.59] | 78% | < .00001 |
| Nishida 2020 | 3.07 [2.21, 4.25] | 78% | < .00001 |
| Shimazaki 2020 | 2.97 [2.27, 3.88] | 76% | < .00001 |
| Wang 2020 | 3.19 [2.41, 4.21] | 79% | < .00001 |
| Nishida 2021 | 3.22 [2.46, 4.22] | 79% | < .00001 |
| Albani 2021 (Newcastle 85+ Study) | 3.41 [2.60, 4.48] | 76% | < .0001 |
| Albani 2021 (TOOTH Study) | 3.50 [2.72, 4.51] | 74% | < .0001 |
| Two studies excluded | | | |
| Gonzalez-Fernandez 2014 and Tanaka 2018 | 3.67 [2.89, 4.64] | 69% | .0006 |
| Gonzalez-Fernandez 2014 and Cohen 2020 | 3.21 [2.16, 4.77] | 80% | < .00001 |
| Cohen 2020 and Tanaka 2018 | 3.50 [2.45, 5.00] | 70% | .0004 |
| Albani 2021 (Newcastle 85+ Study) and Albani 2021 (TOOTH Study) | 3.75 [2.89, 4.87] | 71% | .0003 |
| Albani 2021 (Newcastle 85+ Study) and Tanaka 2018 | 3.91 [3.10, 4.93] | 60% | .008 |
| Albani 2021 (TOOTH Study) and Tanaka 2018 | 3.96 [3.22, 4.85] | 54% | .02 |
| Three studies excluded | | | |
| Albani 2021 (Newcastle 85+ Study), Albani 2021 (TOOTH Study) and Tanaka 2018 | 4.28 [3.60, 5.09] | 33% | .16 |
| Four studies excluded | | | |
| Albani 2021 (Newcastle 85+ Study), Albani 2021 (TOOTH Study), Tanaka 2018 and Gonzalez-Fernandez 2014 | 4.34 [3.64, 5.18] | 37% | .14 |
| Albani 2021 (Newcastle 85+ Study), Albani 2021 (TOOTH Study), Tanaka 2018 and Cohen 2020 | 4.70 [3.85, 5.74] | 4% | .40 |
| Five studies excluded | | | |
| Albani 2021 (Newcastle 85+ Study), Albani 2021 (TOOTH Study), Tanaka 2018, Cohen 2020 and Gonzalez-Fernandez 2014 | 4.77 [3.97, 5.74] | 0% | .44 |

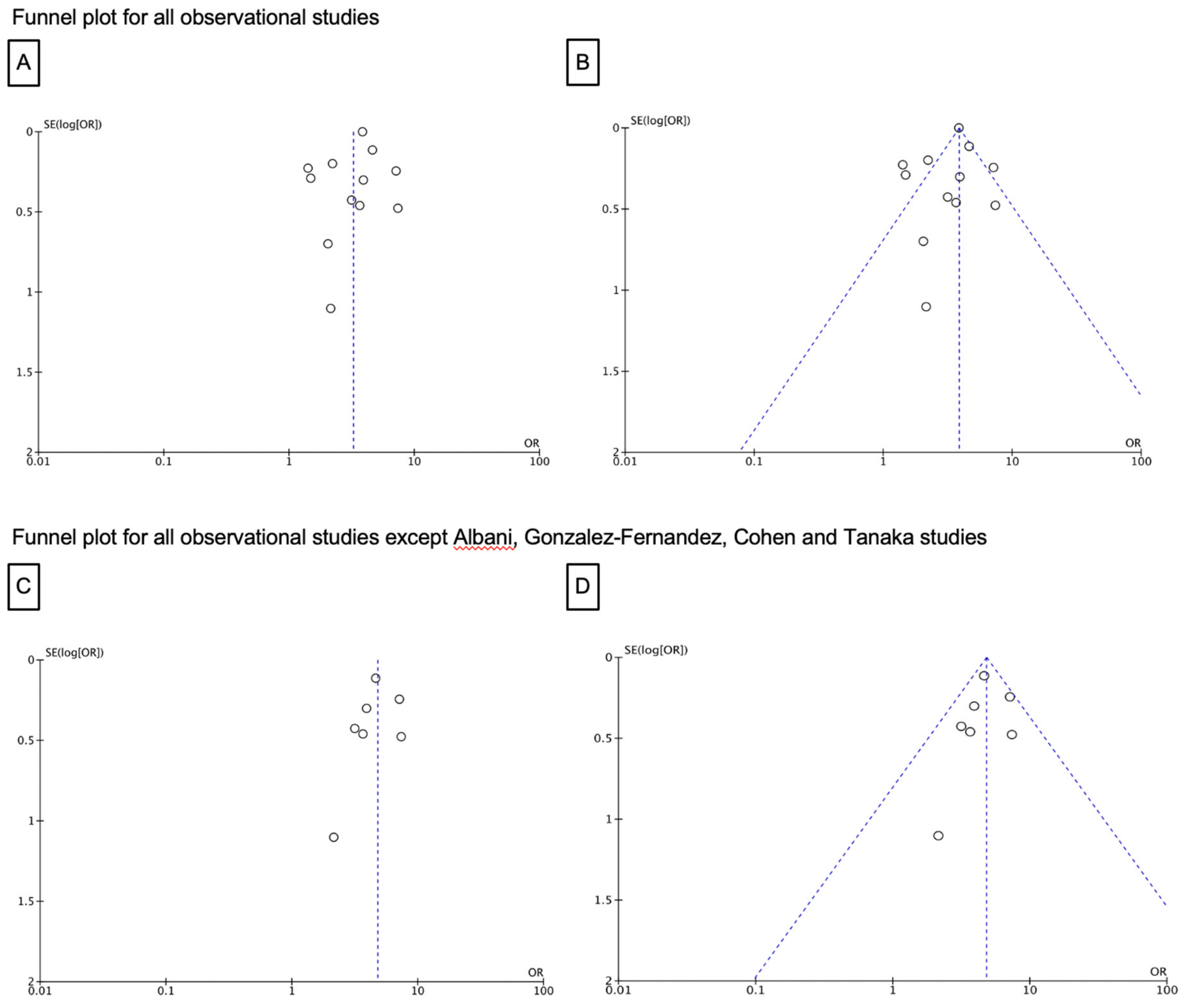


**Figure S1. Funnel plots showing potential publication bias.** (A). Funnel plots for all 12 observational studies; (B). Funnel plots of (A) showing 95% confidence interval (dotted oblique line); (C). Funnel plots for all observational studies except Albani, Gonzalez-Fernandez, Cohen, and Tanaka studies; (D). Funnel plots of (C) showing 95% confidence interval (dotted oblique line).

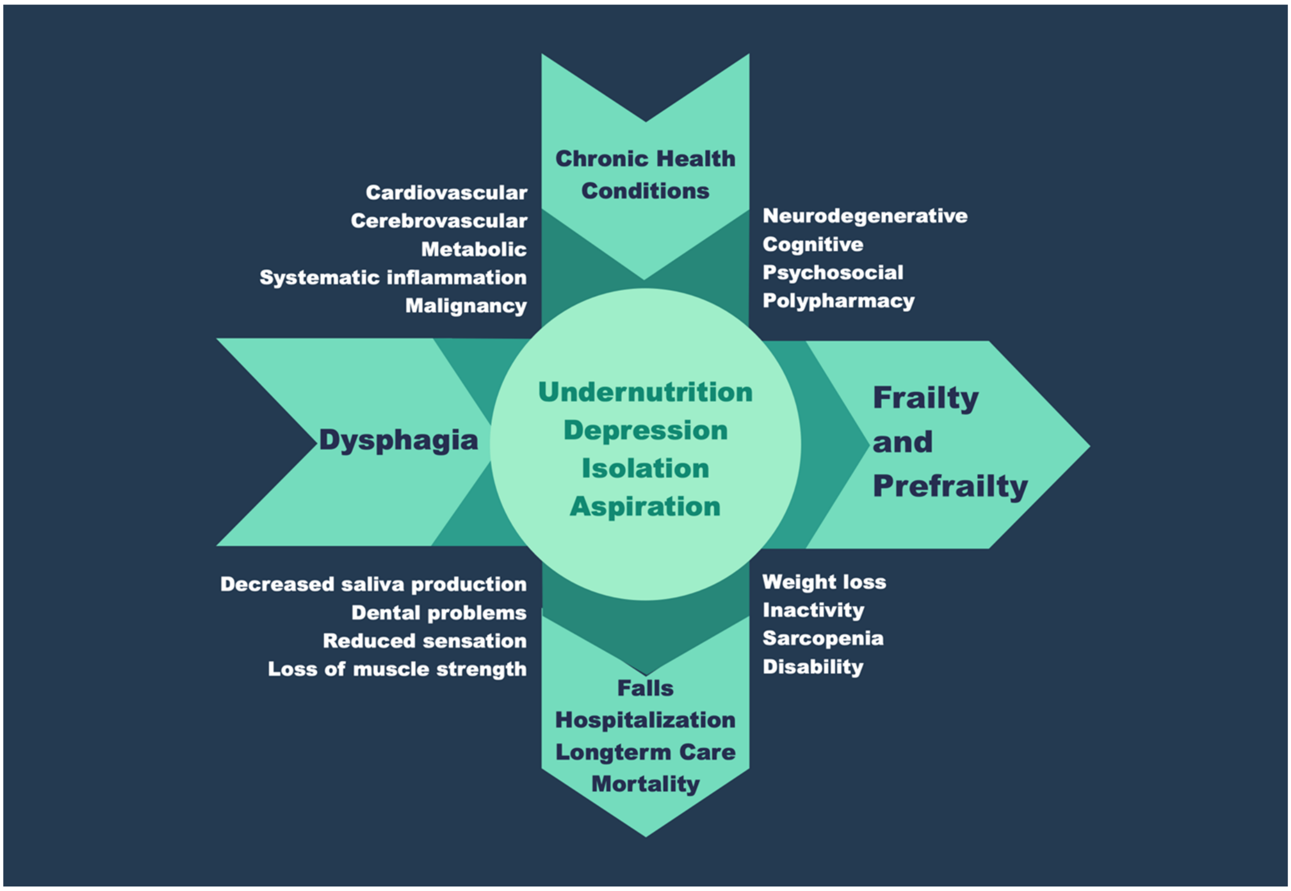


**Figure S2. Illustration of potential linkage between dysphagia and frailty.**

**Methods S1. Detailed Search Strategy**

We systematically searched three databases, namely PubMed, Embase, and Cochrane Database of Systematic Reviews. We used free text search first and then used MeSH terms. The search results were presented as follows.

PubMed

Initial search performed on 25 November 2020
Follow-up search performed on 20 April 2022
No limits applied

| # | Search Term | No. of Results |
|---|---|---|
| 1 | "swallow"[Title/Abstract] OR "swallowing"[Title/Abstract] OR "absorb"[Title/Abstract] OR "devour"[Title/Abstract] OR "drink"[Title/Abstract] OR "eat"[Title/Abstract] OR "gobble"[Title/Abstract] OR "gulp"[Title/Abstract] OR "ingest"[Title/Abstract] OR "wash down"[Title/Abstract] | 90 089 |
| 2 | "disturbance"[Title/Abstract] OR "loss"[Title/Abstract] OR "impairment"[Title/Abstract] OR "dysfunction"[Title/Abstract] OR "decline"[Title/Abstract] OR "reduc*"[Title/Abstract] OR "decreas*"[Title/Abstract] OR "diminish*"[Title/Abstract] OR "difficult*"[Title/Abstract] OR "problem*"[Title/Abstract] OR "trouble"[Title/Abstract] OR "issues"[Title/Abstract] OR "deficit*"[Title/Abstract] OR "deficien*"[Title/Abstract] OR "insufficien*"[Title/Abstract] OR "hard"[Title/Abstract] OR "poor"[Title/Abstract] OR "bad"[Title/Abstract] OR "low"[Title/Abstract] | 11 1262 132 |
| 3 | 1 AND 2 | 49 921 |
| 4 | "dysphagia"[All Fields] OR "deglutition disorders"[All Fields] | 42 054 |
| 5 | 3 OR 4 | 84 909 |
| 6 | "old"[Title/Abstract] OR "old adult"[Title/Abstract] OR "aged"[Title/Abstract] OR "aging"[Title/Abstract] OR "elder"[Title/Abstract] OR "elderly"[Title/Abstract] | 2 104 711 |
| 7 | 5 AND 6 | 15 732 |
| 8 | "frailty"[All Fields] | 21 846 |
| 9 | 7 AND 8 | 117 |

Controlled Vocabulary: Medical Subject Headings (MeSH)

Initial search performed on 25 November 2020
Follow-up search performed on 20 April 2022
No limits applied

| # | Search Term | No. of Results |
|---|---|---|
| 1 | "deglutition disorders"[MeSH] AND "frailty"[MeSH] | 19 |
| 2 | "deglutition disorders"[MeSH] AND "frailty"[MeSH] AND ("aged"[MeSH] OR "aging"[MeSH]) | 15 |

<u>Embase</u>

Initial search performed on 25 November 2020
Follow-up search performed on 20 April 2022

| # | Search Term | No. of Results |
|---|---|---|
| 1 | ("dysphagia" OR "deglutition disorders") AND ("old" OR "old adult" OR "aged" OR "aging" OR "elder" OR "elderly") AND "frailty" | 235 |

<u>Cochrane Database of Systematic Reviews</u>

Initial search performed on 25 November 2020
Follow-up search performed on 20 April 2022
Word variations excluded (the default function in advanced search, for greater precision)
No other limits applied

| # | Search Term | No. of Results |
|---|---|---|
| 1 | "swallow" OR "swallowing" OR "absorb" OR "devour" OR "drink" OR "eat" OR "gobble" OR "gulp" OR "ingest" OR "wash down" | 18 737 |
| 2 | "disturbance" OR "loss" OR "impairment" OR "dysfunction" OR "decline" OR "reduc*" OR "decreas*" OR "diminish*" OR "difficult*" OR "problem*" OR "trouble" OR "issues" OR "deficit*" OR "deficien*" OR "insufficien*" OR "hard" OR "poor" OR "bad" OR "low" | 472 430 |
| 3 | 1 AND 2 | 7 131 |
| 4 | "dysphagia" OR "deglutition disorders" | 5 176 |
| 5 | 3 OR 4 | 11 639 |

| | | |
|---|---|---|
| 6 | "old" OR "old adult" OR "aged" OR "aging" OR "elder" OR "elderly" | 638 414 |
| 7 | 5 AND 6 | 5 400 |
| 8 | "frailty" | 2 555 |
| 9 | 7 AND 8 | 39 |

Controlled Vocabulary: Medical Subject Headings (MeSH)
Initial search performed on 25 November 2020
No limits applied

| # | Search Term | No. of Results |
|---|---|---|
| 1 | Deglutition disorders explode all trees | 18 |
| 2 | Frailty explode all trees | 51 |
| 3 | "aged" OR "aging" | 555 920 |
| 4 | 1 AND 2 AND 3 | 1 |

## Methods S2. MOOSE checklist

MOOSE (Meta-analyses Of Observational Studies in Epidemiology) Checklist

A reporting checklist for Authors, Editors, and Reviewers of Meta-analyses of Observational Studies. You must report the page number in your manuscript where you consider each of the items listed in this checklist. If you have not included this information, either revise your manuscript accordingly before submitting or note N/A.

| Reporting Criteria | Reported (Yes/No) |
|---|---|
| **Reporting of Background** | |
| Problem definition | YES |
| Hypothesis statement | YES |
| Description of Study Outcome(s) | YES |
| Type of exposure or intervention used | YES |
| Type of study design used | YES |
| Study population | YES |
| **Reporting of Search Strategy** | |
| Qualifications of searchers (eg, librarians and investigators) | YES |
| Search strategy, including time period included in the synthesis and keywords | YES |
| Effort to include all available studies, including contact with authors | YES |
| Databases and registries searched | YES |
| Search software used, name and version, including special features used (eg, explosion) | YES |
| Use of hand searching (eg, reference lists of obtained articles) | YES |
| List of citations located and those excluded, including justification | YES |
| Method for addressing articles published in languages other than English | YES |
| Method of handling abstracts and unpublished studies | YES |
| Description of any contact with authors | YES |
| **Reporting of methods** | |
| Description of relevance or appropriateness of studies assembled for assessing the hypothesis to be tested | YES |
| Rationale for the selection and coding of data (eg, sound clinical principles or convenience) | YES |

| Reporting Criteria | Reported (Yes/No) |
|---|---|
| Documentation of how data were classified and coded (eg, multiple raters, blinding, and interrater reliability) | YES |
| Assessment of confounding (eg, comparability of cases and controls in studies where appropriate) | YES |
| Assessment of study quality, including blinding of quality assessors; stratification or regression on possible predictors of study results | YES |
| Assessment of heterogeneity | YES |
| Description of statistical methods (eg, complete description of fixed or random effects models, justification of whether the chosen models account for predictors of study results, dose-response models, or cumulative meta-analysis) in sufficient detail to be replicated | YES |
| Provision of appropriate tables and graphics | YES |
| **Reporting of Results** | |
| Table giving descriptive information for each study included | YES |
| Results of sensitivity testing (eg, subgroup analysis) | YES |
| Indication of statistical uncertainty of findings | YES |
| **Reporting of Discussion** | |
| Quantitative assessment of bias (eg, publication bias) | YES |
| Justification for exclusion (eg, exclusion of non-English-language citations) | YES |
| Assessment of quality of included studies | YES |
| **Reporting of Conclusions** | |
| Consideration of alternative explanations for observed results | YES |
| Generalization of the conclusions (ie, appropriate for the data presented and within the domain of the literature review) | YES |
| Guidelines for future research | YES |
| Disclosure of funding source | YES |

Once you have completed this checklist, please save a copy and upload it as part of your submission. DO NOT include this checklist as part of the main manuscript document. It must be uploaded as a separate file.

**Protocol**

# Protocol for a systematic review and meta-analysis on the associations between dysphagia and frailty and prefrailty outcomes in older adults

Ru-Yung Yang[1*], An-Yun Yang[2,3*], Yong-Chen Chen[3,4*], Shyh-Dye Lee[5,6], Shao-Huai Lee[7], Jeng-Wen Chen[2,3,8,9]

[1]Graduate Institute of Business Administration, Fu Jen Catholic University, New Taipei City, Taiwan
[2]Department of Otolaryngology–Head and Neck Surgery, Cardinal Tien Hospital and School of Medicine, Fu Jen Catholic University, New Taipei City, Taiwan
[3]Master Program of Big Data in Biomedicine, School of Medicine, Fu Jen Catholic University, New Taipei City, Taiwan
[4]College of Medicine, School of Medicine, Fu Jen Catholic University, New Taipei City, Taiwan
[5]Fu Jen Affiliated Clinics, Fu Jen Catholic University Hospital, New Taipei City, Taiwan
[6]Graduate Program of Long-Term (Custodial) Care, School of Medicine, Fu Jen Catholic University, New Taipei City, Taiwan
[7]Department of Oral Hygiene and Healthcare, Cardinal Tien Junior College of Healthcare and Management, New Taipei City, Taiwan
[8]Department of Medical Education and Research, Cardinal Tien Hospital, New Taipei City, Taiwan
[9]Department of Otolaryngology–Head and Neck Surgery, National Taiwan University Hospital, Taipei City, Taiwan

Corresponding Author:
Dr. Jeng-Wen Chen,
Cardinal Tien Hospital and School of Medicine, Fu Jen Catholic University
Director, Otolaryngology–Head and Neck Surgery
Deputy Director, Medical Education and Research
362, Zhongzheng Road, Xindian Dist., New Taipei City, Taiwan 23148
Tel.: +886-2-22194369; Fax: +886-2-22198442
Email: 086365@mail.fju.edu.tw

No existing protocols are currently available for this topic. This protocol is not registered. This work is investigator-initiated. The corresponding author received funding from the Ministry of Science and Technology of the Republic of China (Taiwan) under grant MOST 109-2511-H-567-001-MY2 and, in part, funded by Cardinal Tien Hospital under grant CTH109A-2207 and CTH110A-2202. The funders had no role in the design and conduct of the study; collection, management, analysis, and interpretation of the data; preparation, review, or approval of the manuscript; and decision to submit the manuscript for publication.

## Introduction

Aging is an ongoing global health concern. The total number of people aged ≥60 years worldwide is estimated to rise from approximately 1 billion in 2020 to 2 billion by 2050.[1] Notably, according to experts, the older-age dependency ratio, defined as the number of people aged ≥65 years per 100 working-age adults (aged 20-64 years), is projected to nearly double from 17 in 2020 to 30 in 2050; this signifies that the ratio of working-age adults to older persons will drop to only 3:1.[2] To address this trend, the World Health Organization initiated an action plan in 2020 to improve functional ability, intrinsic capacity, and environment, which are considered crucial components for achieving the goal of "healthy aging."[3] Of these components, optimizing functional ability in older adults is the most effective for achieving independent and healthy aging.

Safe and effective swallowing is a bodily function essential to human life. Safe swallowing involves an alternating on-and-off interaction between respiration and swallowing and an integrated cough reflex.[4,5] Effective swallowing also requires intact muscular and neurocognitive coordination. However, these life-sustaining functions decline with advanced ages.[6] Strategies for adapting to these hazardous aging-related changes, including modifications to food consistency, adoption of swallow postures and maneuvers, and cortical compensation,[7] may help minimize the effect of such changes on quality of life; nevertheless, the accumulation of precipitating factors (eg, decreased saliva production; worsening dental problems; reduced oral, pharyngeal, and esophageal mucosal sensitivity; and loss of muscle mass and strength) may lead to decompensation that engenders an increased susceptibility to swallowing dysfunction.[8] Consequently, swallowing difficulties manifest with aging.[9,10]

Dysphagia is a subjective feeling of difficulty or discomfort in safely and effectively moving a dietary bolus from the oral cavity to the stomach. The difficulty or discomfort may involve the passage of the bolus from the oral cavity to the esophagus (ie, oropharyngeal dysphagia), passage of the bolus from the esophagus to the stomach (ie, esophageal dysphagia), or both. The prevalence of dysphagia increases with advanced age.[11] Using submental surface electromyography and nasal airflow measurement, Wang et al[12] demonstrated that compared with the young- and middle-aged participants, healthy older community-dwelling participants had a significantly delayed onset of swallowing, a longer

duration of swallowing apnea, and a greater probability of piecemeal deglutition. In individuals aged younger than 60 years, dysphagia is usually associated with oncologic and neurologic pathologies, but in older individuals, it is related to the aging process alone or engendered by neurological and neurodegenerative comorbidities.[11,13] Dysphagia is highly prevalent in different cohorts of older people,[14] including community-dwelling individuals (11.4%-33.7%), facility-dwelling individuals (38%-51%), hospitalized individuals (29.4%-47%), and hospitalized individuals with community-acquired pneumonia (55%-91.7%), compared with the general population. Because dysphagia is a multifactorial disorder that is caused by multiple etiological factors, the relationship between dysphagia and the aging process or geriatric syndromes such as frailty, regardless of other comorbidities, remains a focus of research.

In clinical geriatric medicine, frailty typically refers to progressive multisystem dysfunction and increasing vulnerability. The American version of the *International Classification of Diseases*, *Tenth Revision, Clinical Modification* includes the diagnostic code R54 for reimbursement claims effective from October 1, 2020; this code is currently applicable for defining clinical entities not otherwise specified, including frailty, old age, senescence, senile asthenia, and senile debility, except for age-related cognitive decline, sarcopenia, senile psychosis, and senility. Because of the lack of a rigid set of aging biomarkers and the variance in the onset time of the transition from dysfunction to disease among elderly people, controversy remains about whether aging represents a disease that should be included in the *upcoming International Classification of Diseases, Eleventh Revision, Clinical Modification* diagnosis codes. Frailty is characterized by a declining physiological reserve and loss of resistance to minor internal or external stressors caused by cumulative age-related functional deficits.[15] Fried et al[16] described 5 elements of a frailty phenotype: low strength, slow motor performance, exhaustion, low physical activity, and recent unintentional weight loss. Dysphagia and frailty share the characteristics of a geriatric syndrome because they are highly prevalent among older adults;[17,18] are caused by multiple factors; are associated with several comorbidities; predict poor clinical outcomes such as falls,[19] disability,[20] hospitalization,[21] long-term care institutionalization, and mortality,[15,22] and require a multidisciplinary approach for their imrpovement.[13] Therefore, recognizing amendable factors associated with the development of frailty is a crucial aspect of aging care.

Bahat et al.[8] reported an independent association of dysphagia with frailty scores—regardless of age, existence of neurodegenerative disorders, number of chronic diseases, or polypharmacy—in older adults selected from a geriatric outpatient clinic. Researchers have reported a strong association between deteriorated swallowing function and frailty,[23,24] and an increasing number of studies have suggested that dysphagia is a risk factor for frailty in older adults;[8,23-29] nevertheless, the findings of epidemiologic research on the connections between dysphagia and frailty have been inconclusive.[30-33] Integrated studies investigating the association between dysphagia and frailty are inadequate.

Moreover, whether dysphagia disorders in older people living in different settings, which could have multiple etiologies, are risk factors for frailty is unclear. Accordingly, we conducted a systematic review and meta-analysis of cross-sectional and longitudinal studies to substantiate the potential associations between dysphagia and frailty. We hypothesized that dysphagia in older adults may demonstrate an increased risk of frailty-related phenomena, regardless of phenotype. Clarifying these associations might provide new perspectives for delaying the development of frailty in older adults.

**Methods**

This protocol was written with reference to the Meta-Analysis of Observational Studies in Epidemiology (MOOSE) checklist. Ethical approval is not required as we will only conduct a secondary analysis of available data published in the literature. We will submit for peer-review publication and conference presentations after completion of the systematic review and meta-analysis.

**Eligibility Criteria**

Inclusion criteria:

1. Population: adults aged ≥50 years, including community-dwelling, residential care institutions or residential facilities dwelling and hospitalized old adults.
2. Exposures: impairment of function of swallowing, deglutition disorders or dysphagia, measured using objective or validated subjective assessments, as well as self-report.
3. Comparators: participants without impairments of function of swallowing, deglutition disorders or dysphagia as defined above.
4. Outcomes: prevalence, incidence or progression of pre-frailty and frailty, defined based on original or modified versions of validated criteria. For example, the Fried frailty phenotype defines pre-frailty as the presence of 1-2, and frailty as ≥3 of the following 5 criteria:
    (1) unintentional weight loss (10 lbs in past year)
    (2) self-reported exhaustion
    (3) weakness (grip strength)
    (4) slow walking speed
    (5) low physical activity

However, we will accept other definitions of frailty and discuss the limitations of this approach.

5. Study type: observational studies (cross-sectional, longitudinal, and case-control) published as full-length articles or conference abstracts in peer-reviewed journals.

Exclusion criteria:

1. Specific sub-populations/special risk groups (e.g. individuals with cardiovascular disease or with dementia).

2. Presence of impairments of swallowing function in the definition of frailty.
3. Disease-specific instead of motor function-specific or non-motor function-specific associations (e.g. stroke, brain tumors, dementia, depression, head and neck cancers, irradiations, esophageal cancer or other gastrointestinal disorders that will cause impaired swallowing function, psychiatric disorders).

We will not limit the searches by historical time constraints or language.

**Information Sources**

We will systematically search 3 databases (PubMed, Embase and the Cochrane Database of Systematic Reviews). We will also hand-search the reference lists of the included articles and relevant reviews or journals, where applicable. We will accept published poster abstracts if they are indexed in the above databases. We will attempt contact with the corresponding authors to obtain additional unpublished information such as crude number of participants that was stratified by dysphagia and frailty development and covariate-adjusted effect estimates.

**Search Strategy**

We will search PubMed, Embase and Cochrane using the following search strategy:

Free text

| # | Search Term |
|---|---|
| 1 | "swallow"[Title/Abstract] OR "swallowing"[Title/Abstract] OR "absorb"[Title/Abstract] OR "devour"[Title/Abstract] OR "drink"[Title/Abstract] OR "eat"[Title/Abstract] OR "gobble"[Title/Abstract] OR "gulp"[Title/Abstract] OR "ingest"[Title/Abstract] OR "wash down"[Title/Abstract] |
| 2 | "disturbance"[Title/Abstract] OR "loss"[Title/Abstract] OR "impairment"[Title/Abstract] OR "dysfunction"[Title/Abstract] OR "decline"[Title/Abstract] OR "reduc*"[Title/Abstract] OR "decreas*"[Title/Abstract] OR "diminish*"[Title/Abstract] OR "difficult*"[Title/Abstract] OR "problem*"[Title/Abstract] OR "trouble"[Title/Abstract] OR "issues"[Title/Abstract] OR "deficit*"[Title/Abstract] OR "deficien*"[Title/Abstract] OR "insufficien*"[Title/Abstract] OR "hard"[Title/Abstract] OR "poor"[Title/Abstract] OR "bad"[Title/Abstract] OR "low"[Title/Abstract] |
| 3 | 1 AND 2 |
| 4 | "dysphagia"[All Fields] OR "deglutition disorders"[All Fields] |
| 5 | 3 OR 4 |
| 6 | "old"[Title/Abstract] OR "old adult"[Title/Abstract] OR "aged"[Title/Abstract] OR "aging"[Title/Abstract] OR "elder"[Title/Abstract] OR "elderly"[Title/Abstract] |
| 7 | 5 AND 6 |
| 8 | "frailty"[All Fields] |

| | |
|---|---|
| 9 | 7 AND 8 |

Controlled Vocabulary: Medical Subject Headings (MeSH)
No limits applied

| # | Search Term |
|---|---|
| 1 | "deglutition disorders"[MeSH] AND "frailty"[MeSH] |
| 2 | "deglutition disorders"[MeSH] AND "frailty"[MeSH] AND ("aged"[MeSH] OR "aging"[MeSH]) |

**Data Management**

We will export the search results to EndNote to remove duplicates and manually screen the records.

**Selection Process**

We will screen potentially eligible studies based on title and abstract, following which, we will retrieve full texts for evaluation. This will be done by 2 independent reviewers.

**Data Extraction & Data Items**

We will extract data from each article into a standardized extraction template by using Review Manager (RevMan), Version 5.4 (The Cochrane Collaboration, London, United Kingdom). This data will include: first author, year published, study design, setting, country, region, sample size, percentage female, mean or median age, method of identifying dysphagia, frailty definition, and adjustment for confounders. One reviewer will extract the data, and this will be verified by at least 1 other reviewer.

**Risk of Bias**

We plan to use the Newcastle-Ottawa Scale (NOS), to evaluate the risk of bias at the outcome level. As per the NOS grading in past reviews, we will grade studies as having a high (<5 stars), moderate (5-7 stars) or low risk of bias (≥8 star). For longitudinal studies, the NOS assigns a score out of 9 to each study (where 9 indicates that the study meets all 9 criteria for quality assessments and 0 indicates that the study does not meet any of the criteria) based on potential domains of bias, such as selection, comparability, and outcome. For cross-sectional studies, the modified NOS[34] assigns a score out of 10 to each study.

**Statistical Analysis**

We will calculate pooled ORs by conducting a fixed-effects or random-effects meta-analysis of raw data extracted from each study. The precision levels of the effect sizes will be expressed as 95% CIs. We will assess the statistical heterogeneity and inconsistency of the effects across the included studies by using the Cochran Q test ($P < .10$) and $I^2$

statistics, respectively. We will perform subsequent subgroup analyses by stratifying the data by demographics, study design, and study population to characterize potential sources of heterogeneity. In the subgroup analyses, we will calculate the risk estimates for outcomes of frailty, prefrailty or frailty and prefrailty combined. Additionally, we will conduct sensitivity analyses by sequentially excluding individual studies to examine the effect of such exclusions on the overall risk estimate. Publication bias was evaluated visually through funnel plots. A 2-sided *P* value of <.05 was deemed statistically significant.

**Keywords**
deglutition disorders, dysphagia, aging, frailty, community-dwelling, residential facility dwelling

**References**

1. World Health Organization. Ageing: Overview. 2021. Accessed November 1, 2020. https://www.who.int/health-topics/ageing#tab=tab_1
2. United States Census Bureau. Demographic trends of global aging. An Aging World: 2020. November 1, 2020. https://mtgis-portal.geo.census.gov/arcgis/apps/MapSeries/index.html?appid=3d832796999042daae7982ff36835e2e
3. World Health Organization. Decade of healthy ageing: baseline report. 2021. Accessed November 1, 2020. https://www.who.int/publications/i/item/9789240017900
4. Martin-Harris B. Clinical implications of respiratory-swallowing interactions. *Curr Opin Otolaryngol Head Neck Surg.* 2008;16(3):194-199. doi:10.1097/MOO.0b013e3282febd4b
5. Valenzano TJ, Guida BT, Peladeau-Pigeon M, Steele CM. Respiratory-Swallow Coordination in Healthy Adults During Drinking of Thin to Extremely Thick Liquids: A Research Note. *J Speech Lang Hear Res.* 2020;63(3):702-709. doi:10.1044/2019_JSLHR-19-00163
6. Humbert IA, Fitzgerald ME, McLaren DG, et al. Neurophysiology of swallowing: effects of age and bolus type. *Neuroimage.* 2009;44(3):982-991. doi:10.1016/j.neuroimage.2008.10.012
7. Teismann IK, Steinstraeter O, Schwindt W, Ringelstein EB, Pantev C, Dziewas R. Age-related changes in cortical swallowing processing. *Neurobiol Aging.* 2010;31(6):1044-1050. doi:10.1016/j.neurobiolaging.2008.07.001
8. Bahat G, Yilmaz O, Durmazoglu S, Kilic C, Tascioglu C, Karan MA. Association between Dysphagia and Frailty in Community Dwelling Older Adults. *J Nutr Health Aging.* 2019;23(6):571-577. doi:10.1007/s12603-019-1191-0
9. Robbins J, Hamilton JW, Lof GL, Kempster GB. Oropharyngeal swallowing in normal adults of different ages. *Gastroenterology.* 1992;103(3):823-829. doi:10.1016/0016-5085(92)90013-o

10. Muhle P, Wirth R, Glahn J, Dziewas R. [Age-related changes in swallowing. Physiology and pathophysiology]. *Nervenarzt.* 2015;86(4):440-451. doi:10.1007/s00115-014-4183-7
11. Ney DM, Weiss JM, Kind AJ, Robbins J. Senescent swallowing: impact, strategies, and interventions. *Nutr Clin Pract.* 2009;24(3):395-413. doi:10.1177/0884533609332005
12. Wang CM, Chen JY, Chuang CC, Tseng WC, Wong AM, Pei YC. Aging-related changes in swallowing, and in the coordination of swallowing and respiration determined by novel non-invasive measurement techniques. *Geriatr Gerontol Int.* 2015;15(6):736-744. doi:10.1111/ggi.12343
13. Ortega O, Martin A, Clave P. Diagnosis and Management of Oropharyngeal Dysphagia Among Older Persons, State of the Art. *J Am Med Dir Assoc.* 2017;18(7):576-582. doi:10.1016/j.jamda.2017.02.015
14. Baijens LW, Clave P, Cras P, et al. European Society for Swallowing Disorders - European Union Geriatric Medicine Society white paper: oropharyngeal dysphagia as a geriatric syndrome. *Clin Interv Aging.* 2016;11:1403-1428. doi:10.2147/CIA.S107750
15. Clegg A, Young J, Iliffe S, Rikkert MO, Rockwood K. Frailty in elderly people. *Lancet.* 2013;381(9868):752-762. doi:10.1016/S0140-6736(12)62167-9
16. Fried LP, Tangen CM, Walston J, et al. Frailty in older adults: evidence for a phenotype. *J Gerontol A Biol Sci Med Sci.* 2001;56(3):M146-156. doi:10.1093/gerona/56.3.m146
17. Ofori-Asenso R, Chin KL, Mazidi M, et al. Global Incidence of Frailty and Prefrailty Among Community-Dwelling Older Adults: A Systematic Review and Meta-analysis. *JAMA Netw Open.* 2019;2(8):e198398. doi:10.1001/jamanetworkopen.2019.8398
18. Achem SR, Devault KR. Dysphagia in aging. *J Clin Gastroenterol.* 2005;39(5):357-371. doi:10.1097/01.mcg.0000159272.88974.54
19. Hatcher VH, Galet C, Lilienthal M, Skeete DA, Romanowski KS. Association of Clinical Frailty Scores With Hospital Readmission for Falls After Index Admission for Trauma-Related Injury. *JAMA Netw Open.* 2019;2(10):e1912409. doi:10.1001/jamanetworkopen.2019.12409
20. Gill TM, Allore HG, Gahbauer EA, Murphy TE. Change in disability after hospitalization or restricted activity in older persons. *JAMA.* 2010;304(17):1919-1928. doi:10.1001/jama.2010.1568
21. Gill TM, Allore HG, Holford TR, Guo Z. Hospitalization, restricted activity, and the development of disability among older persons. *JAMA.* 2004;292(17):2115-2124. doi:10.1001/jama.292.17.2115
22. Wei K, Nyunt MS, Gao Q, Wee SL, Yap KB, Ng TP. Association of Frailty and Malnutrition With Long-term Functional and Mortality Outcomes Among Community-Dwelling Older Adults: Results From the Singapore Longitudinal Aging Study 1. *JAMA Netw Open.* 2018;1(3):e180650. doi:10.1001/jamanetworkopen.2018.0650

23. Shimazaki Y, Nonoyama T, Tsushita K, Arai H, Matsushita K, Uchibori N. Oral hypofunction and its association with frailty in community-dwelling older people. *Geriatr Gerontol Int.* 2020;20(10):917-926. doi:10.1111/ggi.14015
24. Watanabe K, Umegaki H, Huang CH, et al. Association between dysphagia risk and unplanned hospitalization in older patients receiving home medical care. *Geriatr Gerontol Int.* 2019;19(10):977-981. doi:10.1111/ggi.13753
25. Ambagtsheer RC, Beilby J, Seiboth C, Dent E. Prevalence and associations of frailty in residents of Australian aged care facilities: findings from a retrospective cohort study. *Aging Clin Exp Res.* 2020;32(9):1849-1856. doi:10.1007/s40520-019-01379-0
26. Cohen SM, Lekan D, Risoli T, Jr., et al. Association Between Dysphagia and Inpatient Outcomes Across Frailty Level Among Patients >/= 50 Years of Age. *Dysphagia.* 2020;35(5):787-797. doi:10.1007/s00455-019-10084-z
27. Nishida T, Yamabe K, Honda S. Dysphagia is associated with oral, physical, cognitive and psychological frailty in Japanese community-dwelling elderly persons. *Gerodontology.* 2020;37(2):185-190. doi:10.1111/ger.12455
28. Wang T, Zhao Y, Guo A. Association of swallowing problems with frailty in Chinese hospitalized older patients. *Int J Nurs Sci.* 2020;7(4):408-412. doi:10.1016/j.ijnss.2020.09.005
29. Nishida T, Yamabe K, Honda S. The Influence of Dysphagia on Nutritional and Frailty Status among Community-Dwelling Older Adults. *Nutrients.* 2021;13(2). doi:10.3390/nu13020512
30. Albani V, Nishio K, Ito T, et al. Associations of poor oral health with frailty and physical functioning in the oldest old: results from two studies in England and Japan. *BMC Geriatr.* 2021;21(1):187. doi:10.1186/s12877-021-02081-5
31. Gonzalez-Fernandez M, Humbert I, Winegrad H, Cappola AR, Fried LP. Dysphagia in old-old women: prevalence as determined according to self-report and the 3-ounce water swallowing test. *J Am Geriatr Soc.* 2014;62(4):716-720. doi:10.1111/jgs.12745
32. Chang CI, Chan DC, Kuo KN, Hsiung CA, Chen CY. Prevalence and correlates of geriatric frailty in a northern Taiwan community. *J Formos Med Assoc.* 2011;110(4):247-257. doi:10.1016/S0929-6646(11)60037-5
33. Tanaka T, Takahashi K, Hirano H, et al. Oral Frailty as a Risk Factor for Physical Frailty and Mortality in Community-Dwelling Elderly. *J Gerontol A Biol Sci Med Sci.* 2018;73(12):1661-1667. doi:10.1093/gerona/glx225
34. Herzog R, Alvarez-Pasquin MJ, Diaz C, Del Barrio JL, Estrada JM, Gil A. Are healthcare workers' intentions to vaccinate related to their knowledge, beliefs and attitudes? A systematic review. *BMC Public Health.* 2013;13:154. doi:10.1186/1471-2458-13-154
